# Multiplexed plasma protein classifiers for the diagnosis of age‐related macular degeneration

**DOI:** 10.1002/ctm2.1307

**Published:** 2023-06-14

**Authors:** Hee‐Sung Ahn, Youngju Lee, Hyeong Min Kim, Shinyeong Ju, Seonjeong Lee, Hyeon‐Gyo Jeong, Sang Jun Park, Kyu Hyung Park, Junyeop Lee, Joo Yong Lee, Se Joon Woo, Cheolju Lee

**Affiliations:** ^1^ Chemical and Biological Integrative Research Center Korea Institute of Science and Technology Seoul Republic of Korea; ^2^ Convergence Medicine Research Center, Asan Institute for Life Sciences Asan Medical Center Seoul Republic of Korea; ^3^ RetiMark Co. Ltd Seoul Republic of Korea; ^4^ Department of Ophthalmology Seoul National University Bundang Hospital Seoul National University College of Medicine Gyeonggi‐do Republic of Korea; ^5^ Division of Bio‐Medical Science and Technology, KIST School Korea University of Science and Technology Seoul Republic of Korea; ^6^ Department of Ophthalmology, Asan Medical Center University of Ulsan College of Medicine Seoul Republic of Korea

Dear Editor

In this study, for prediction of age‐related macular degeneration (AMD), we developed an in vitro diagnosis model using clinical information and the mass spectrometry based targeted plasma proteomic results (two risk AMD alleles in CFH [rs800292 and rs1061170^1^], IGFBP2, SELE and THBS1). The model created using 300 samples and validated using 613 independent retrospective cohorts showed 0.744 for AUROC value and 31.5% for positive predictive value (PPV) at 95.6% negative predictive value (NPV) assuming AMD prevalence rate to be 10%.

AMD is a globally prominent cause of vision loss and blindness in elderly people^2^, ^3^ with a global prevalence of 5% in those aged ≥ 45 years and up to 20% in people aged ≥ 75 years^4^ and is difficult to diagnose in its early stages due to the absence of symptoms and discomfort of conventional diagnostics such as fundus examination, and photography. Alternative proteomics technologies are also being studied.^5^ One of which is the blood‐based multiple reaction monitoring‐mass spectrometry (MRM‐MS) assay where the peptides of plasma proteins can be detected for inspection, discovery and validation of plasma protein biomarkers.^6^

We subjected 913 clinical plasma samples to a 7‐plex MRM‐MS assay (Figure 1 and Table S1) that contained three peptides of the corresponding three proteins (IGFBP2, SELE and THBS1) and four CFH variants of two risk alleles (rs800292:p.Val62Ile and rs1061170:p.Tyr402His^1^). Assay evaluations of the seven peptides were successfully characterised in plasma samples following the Clinical Proteomic Tumor Analysis Consortium (CPTAC) guidelines.^7^ The lower limits of quantitation of each peptide were calculated based on the response curves (Figure S1) and were between 1 and 1,000 ng/mL (Table S2). This assay satisfied the criteria for the analytical selectivity analysis (Figure S2). In the analytical stability test, SIS‐spiked plasmas were stored at 4°C and measured by MRM analysis at eight time points (0, 6 and 12 h and days 1, 2, 3, 6 and 8), with an average coefficient of variation of 6.7% for seven peptides, which was within 15% (Table S3).

**FIGURE 1:**
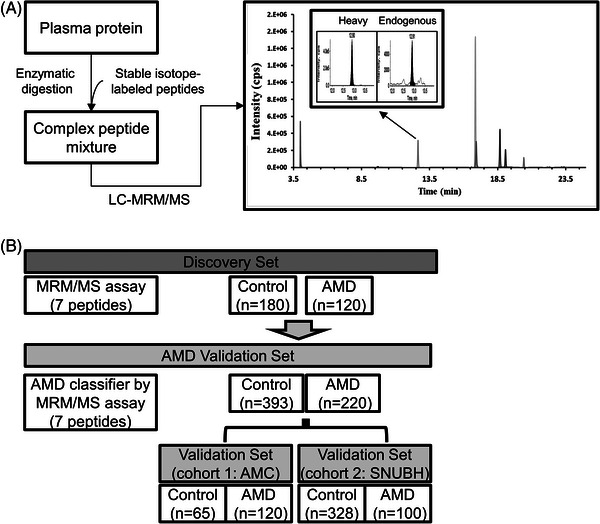
Workflow of stable isotope standard (SIS) diluted multiple reaction monitoring (MRM) assay, building the age‐related macular degeneration (AMD) classifiers using the discovery set, and validating them in the validation sets. (A) Whole plasma was digested by trypsin and spiked SIS peptides. Extracted ion chromatograms of target peptides in liquid chromatography‐multiple reaction monitoring‐mass spectrometry measurement. (B) The AMD classifier using 7‐plex MRM analysis in the discovery set was validated in two cohort sets (AMC and SNUBH). AMC, Asan Medical Center; SNUBH, Seoul National University Bundang Hospital.

In the MRM results of discovery samples, we derived a predictive model using 1000× repeated 10‐fold cross‐validations. We generated protein prediction scores (PPSs) by fitting a support vector machine based on IGFBP2, SELE and THBS1 and subsequently built a logistic regression (LR) model, which included PPS, the two CFH alleles as categorical variables, and clinical factors (i.e. age, BMI, smoking, hypertension, hyperlipidaemia) (Table S4). After building the LR classifiers, we assessed their performance in the discovery set. The AUROC was 0.876 (95% confidence interval [CI], 0.836–0.915; Figure S3). The nominal binary result of the model was transformed into disease prediction scores in the range of 0 to 1 (Figure S4A). We then applied this classifier to an independent validation set. The disease prediction scores for the validation set were calculated (Figure S4B). The AUROC for AMD was 0.744 (95% CI, 0.700–0.787; Figure 2).

**FIGURE 2:**
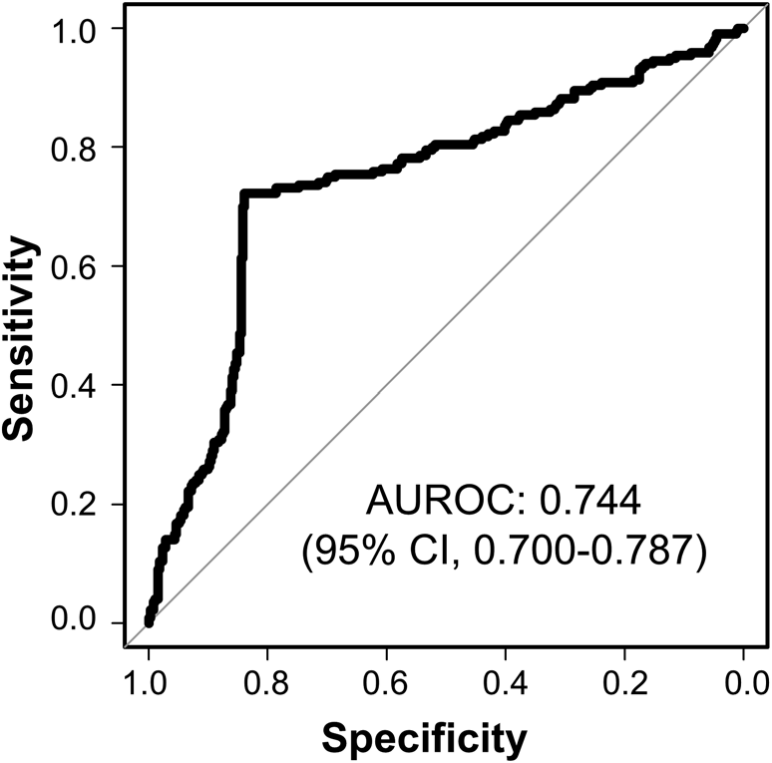
The performance of the age‐related macular degeneration (AMD) classifier in the validation set. The validation set consists of 393 controls and 220 patients with AMD. The area under curve receiver operating characteristic is 0.744 (95% confidence interval: 0.700−0.787) (A). Receiver operating characteristic analysis was executed by the R package (‘pROC’).

PPV and NPV consider the prevalence based on sensitivity and specificity and are important for decision‐making regarding the diagnosis. Therefore, a threshold including PPV or NPV should be established for proper diagnostic testing. We assumed an AMD prevalence of 10% in the elderly population aged > 50 years based on estimates from the literature.^4^ The sensitivity, specificity, PPV and NPV of the classifiers in the discovery set were plotted as functions of the disease prediction scores (Figure 3). A sample was predicted to be normal if its prediction score did not exceed the threshold of AMD. We estimated the classifier's performance in the discovery set assuming the prevalence rates of AMD with a threshold determined by setting the PPVs (AMD = 35%) and ensuring high specificity (<84%). The threshold of the AMD prediction score was 0.44. AMD had 97% NPV, 76.6% sensitivity and 84.4% specificity in the discovery samples, respectively. The threshold selected in the discovery process was applied directly to validation samples consisting of two independent cohorts. At 10% AMD prevalence, PPV, NPV, sensitivity and specificity were 31.5%, 95.6%, 65.5% and 84.2%, respectively, for validation samples. The statistical performances of the thresholds when setting the prevalence to 15% are summarised in Table S5. When the validation results were divided into cohorts of two hospitals (Asan Medical Center [AMC] and Seoul National University Bundang Hospital [SNUBH]), at 10% AMD prevalence, the PPV was 34.4% in the AMC cohort but 29.7% in the SNUBH cohort (Table S6).

**FIGURE 3:**
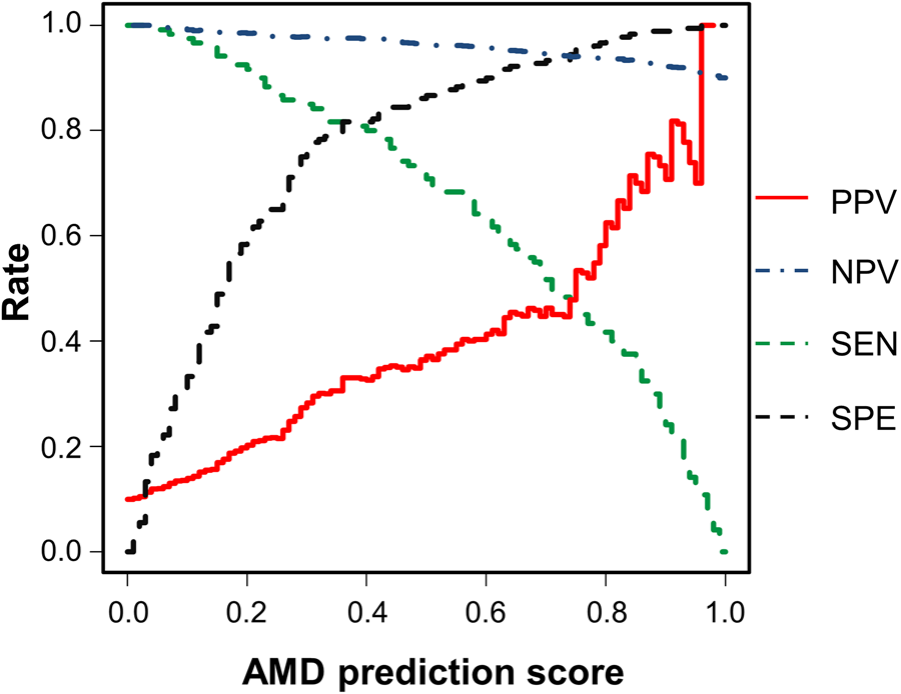
Sensitivity (SEN), specificity (SPE), positive predictive value (PPV) and negative predictive value (NPV) of the classifier at prevalence 10%, depending on the disease predictive scores in the range 0 to 1 on the discovery set. Red line indicates PPV; black dotted line indicates SPE; green dotted line indicates SEN; blue dotted‐dashed line indicates NPV.

The proteins in the MRM panel were clinically and biologically associated with AMD, although not expressed in the eye. SELE and THBS1 play a role in immunoadhesion and mediate blood neutrophils in the cytokine‐activated endothelium,^8^ which can be associated with the mechanism of AMD, and the upstream transcription factors of their proteins were related to hypoxia or inflammatory abnormalities in ocular and retinal diseases (Figure 4A). Blood IGFBP2 is a positive regulator of T‐cell proliferation,^9^ and retinal mRNA expression of *Igfbp2* was demonstrated to increase in aged and lase‐induced choroidal neovascularisation model mouse (Figure S5).

**FIGURE 4:**
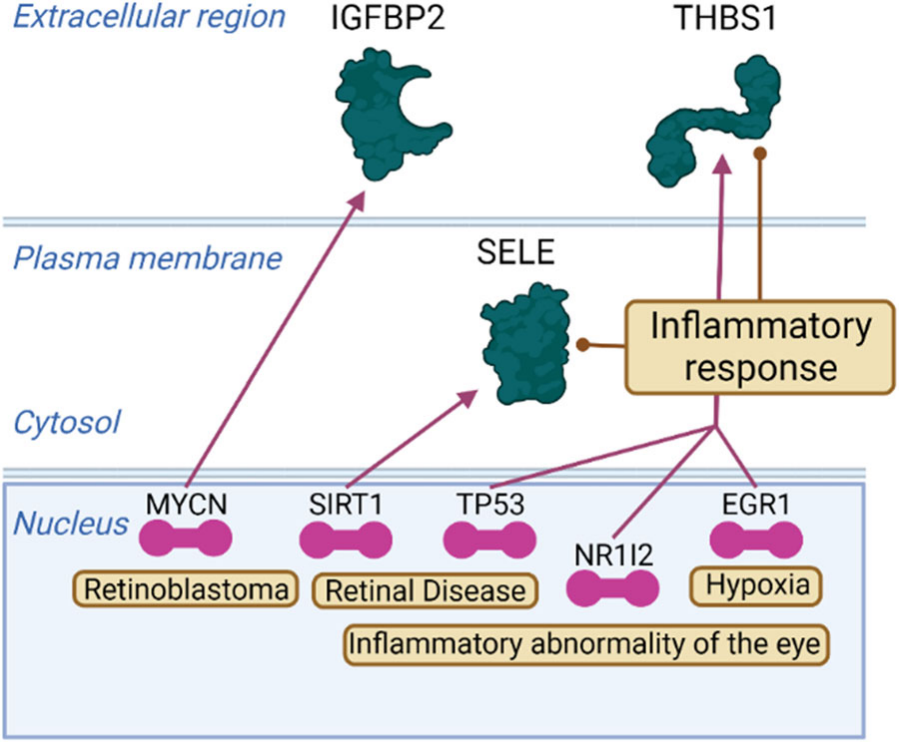
Functional pathway analysis of classifier proteins. The three classifier proteins (green icon), five transcription regulators (pink icon) and the functional terms (brown rounded corner rectangle) of retinal disease, inflammatory abnormality of the eye and hypoxia. Created with BioRender.com.

For optimal visual outcomes, early diagnosis and timely treatment of AMD are important.^3^ By a Markov model, regular screening with fundus examination, although not cost‐effective, is better than oral supplementation for preventing AMD‐induced blindness.^10^ However, still human resources for fundus photography and the patient access to the funduscopic device can be issues in developed and developing countries, and older people are often unwilling to visit the ophthalmology clinic for fundus examination due to reasons such as the distance to the clinic, discomfort associated with fundus examination, medical costs, and lack of knowledge of AMD. Therefore, our simple and convenient screening method based on our plasma proteomics MRM assay that does not require fundus photography and human assessors may help in the early diagnosis and treatment of major retinal disease and may be beneficial in preventing blindness in the elderly populations and revolutionise the current screening method for AMD in the future.

## AUTHOR CONTRIBUTIONS

HS Ahn: interpretation of data, drafting or revising the article; Y Lee: acquisition, analysis and interpretation of data; HM Kim: acquisition of data, analysis and interpretation of data, drafting or revising the article; S Ju: acquisition and analysis of data; S Lee: acquisition and analysis of data; HG Jung, acquisition of data; SJ Park: acquisition of data; KH Park: acquisition of data; J Lee: animal experiment and acquisition of data; JY Lee: acquisition of data; SJ Woo: conception and design, acquisition of data, analysis and interpretation of data, drafting or revising the article; C Lee: conceptual design of the study, analysis and interpretation of data, drafting or revising the article.

## CONFLICT OF INTEREST STATEMENT

HS Ahn: Retimark (O); S Ju: Retimark (O); S Lee: Retimark (O); HG Jung: Retimark (O); KH Park: Retimark (O); SJ Park: Retimark (O); SJ Woo: Samsung Bioepis (C, S), Curacle (C, S), Alteogen (C, S), Novelty Nobility (C, S), Sometech (C), Novartis (C, L, S), Janssen (C), Bayer (L), Allergan (C, L), Abbvie (L, S), Alcon (L), Retimark (O), Panolos Bioscience (O); C Lee: Retimark (O). (C: consultant; L: lecture fee; O: equity owner; S: grant support). The marker proteins in this study were patented by Retimark Co. Ltd (Patent Number PCT/KR2020/003168, PCT/KR/2020/003169, PCT/KR2020/003848)

## DATA SHARING AND DATA ACCESSIBILITY

All MS data were deposited in PASSEL database (accession ID: PASS01756).

## Supporting information

Supplementary Information

Supplementary Information

Supplementary Information

Supplementary Information

Supplementary Information

Supplementary Information

Supplementary Information

Supplementary Information

Supplementary Information

Supplementary Information

Supplementary Information

Supplementary Information
