# Supplementary material for: Multiplexed plasma protein classifiers for the diagnosis of age‐related macular degeneration
Source: Clin Transl Med. 2023 Jun 14;13(6):e1307. doi: 10.1002/ctm2.1307 (PMC10267425; doi:10.1002/ctm2.1307)
Supplement: Supplementary file 1 — Supplementary Information [file CTM2-13-e1307-s004.docx]

**Supplemental methods**

**REAGENTS AND MATERIALS**

Tris(2-carboxyethyl)phosphine (TCEP) and 2-chloroacetamide were purchased from Sigma-Aldrich (St. Louis, MO, USA). Sequencing-grade modified trypsin was obtained from Promega (Madison, WI, USA). High-performance liquid chromatography-grade water and acetonitrile were purchased from J. T. Baker (Radnor, PA, USA). Digested samples were desalted using a Sep-Pak tC18 96-well plate (Waters Corp., Milford, MA, USA). Synthetic stable isotope-labeled standard (SIS) peptides (isotope labeled at the C-terminus of ^13^C_6_ and ^15^N_2_) were synthesized by JPT (Berlin, Germany) (< 95% purity).

**CLINICAL PARTICIPANT INFORMATION AND PLASMA SAMPLES**

The study was approved by the Institutional Review Board of Seoul National University Bundang Hospital (SNUBH) and followed the guidelines of the Declaration of Helsinki (Institutional Review Board [IRB] approval number: B-0801-053-008, B-1708-412-303, B-1810-497-303) and the IRB of Asan Medical Center (AMC, Seoul, Korea; IRB-e no. 2019-0106). Written informed consent was obtained from all the participants.

Patients with age-related macular degeneration (AMD) were recruited from two hospitals (SNUBH and AMC) in South Korea, between January 2008 and January 2014. During the same study period, elderly individuals without AMD (Age-Related Eye Disease Study [AREDS], Category 0 or 1) [^1^](#_ENREF_1) were recruited from the same retina clinic or from among participants recruited for the population-based longitudinal study described in our previous report [^2^](#_ENREF_2). In the discovery set (N = 180), clinical data and plasma samples were collected from 120 patients with AMD and 180 control subjects. For the validation set, 220 patients with AMD and 393 controls (N = 613) were enrolled and analyzed (Table S1).

Peripheral blood was collected from each patient in an EDTA tube and placed on ice for transport to the laboratory where it was centrifuged, aliquoted, and immediately frozen at –80 °C until use [^3^](#_ENREF_3). Plasmas were prepared as suggested by the Human Proteome Organization Plasma Proteome Project [^4^](#_ENREF_4).

**SAMPLE PREPARATION FOR MULTIPLE REACTION MONITORING (MRM)**

Plasma (2 µL) was denatured with 50 µL of 8 M urea in ammonium bicarbonate. The samples were treated with 7 µL of 100 mM TCEP and 7 µL of 200 mM chloroacetamide at 25 °C for 1 h in the dark. The sample was further diluted 10-fold with 50 mM ammonium bicarbonate to bring the urea concentration to less than 1 M. Sequence modified trypsin was added to the sample at an enzyme-to-protein ratio of 1:50 and incubated for 16 h at 37 °C with shaking at 600 rpm. Formic acid was added at a final concentration of 0.5% to stop the digestion reaction. The sample was desalted using Sep-Pak tC18 96-well plate (Waters, Milford, MA, USA), dried using a vacuum concentrator (miVac Duo Concentrator, Genevac, Suffolk, UK), and stored at –80 °C until further use. SIS peptides were spiked into dried samples for MRM analysis [^3^](#_ENREF_3).

**CAPILLARY LIQUID CHROMATOGRAPHY-MRM-MASS SPECTROMETRY (LC-MRM-MS)**

The LC system comprised an ACQUITY UPLC M-class (Waters Corp., Milford, MA, USA). USA) coupled to a 5500 Qtrap mass spectrometer using a turbo v electrospray ion source (SCIEX, Foster City, CA, USA). Samples were reconstituted with 40 μL of stable isotope standard peptide mixture in mobile phase A, injected with a full sample loop volume of 5 μL, and separated in an ACQUITY UPLC peptide CSH C18 column (1 mm i.d., 10 cm length, pore size 130 Å, particle size 1.7 μm; Waters). The step gradient 5% B for 3 min, 5–25% B for 21.5 min, 25–90% for 0.5 min, washing the column 90–90% for 5 min, 90–5% B for 1 min, equilibrate the column with 5% B for 9 min, and with a flow rate of 20 μL/min were applied. LC-MRM was carried out in a positive mode as per the following parameters: ion spray voltage of 5500 V, curtain gas at 20 psi, nebulizer gas at 25 psi, heating gas at 20 psi, resolution at 0.7 Da (unit) for Q1/Q3, interface temperature at 400 °C, and scan mass range QQQ mode of m/z is 300–1250. The declustering potential (DP) and collision energy (CE) of the peptide parameters were optimized using SIS peptides. Quantification experiments were performed using the scheduled MRM mode with a window time of 120 s and a cycle time of 1.5 s. The mass spectrometer was operated using the Analyst software (Version 1.5.2; SCIEX), which generated LC-MRM data (*.wiff) and used to analyze the results from the extracted ion chromatogram.

**RESPONSE CURVE**

Reverse response curves were pooled from 20 plasma and matrix samples to construct a serially diluted SIS peptide mixture of 6–10 concentration points, and LC-MRM was analyzed in triplicate. The lower limit of quantitation (LLOQ), upper limit of quantitation (ULOQ), and linear range of the target peptides were determined by generating reverse response curves. The limit of detection (LOD) is generally used to estimate three times the signal-to-noise ratio (S/N), and the limit of quantification (LOQ) is used to estimate 10 times the signal-to-noise ratio. LLOQ and ULOQ values were determined with the lowest and highest concentration estimates for linearity with coefficients of determination (R^2^) > 0.99 and coefficients of variation (CV) < 20%, respectively [^5-8^](#_ENREF_5).

**QUALITY CONTROL (QC) SAMPLE**

The low-QC samples at two times the LOQ of the response curve range were prepared by spiking the SIS peptide.

**ANALYTICAL SPECIFICITY TEST**

The six double-blank samples did not contain endogenous substances or standards. The blank sample contained only the standard products. Specific analytes are endogenous materials without standards. Low-QC samples were prepared with endogenous digested plasma samples and twice of LOQ of SIS peptides. LC-MRM analysis was performed in the following order: double blank, blank, specificity analytes, and low-QC. These analyses were repeated once for six double blank samples and three replicates for the blank, specificity analytes, and low-QC samples.

**ANALYTICAL STABILITY TEST**

Four individual plasma samples of the spiked SIS peptides were prepared in 14 aliquots. All the samples were analyzed after thawing. Each Seven sets samples were stored at 4 and –80 °C until analysis. The first set of samples (0 h) was analyzed immediately after preparation. MRM analyses were then performed in the order of 6 h, 12 h, 1 d, 2 d, 3 d, 6 d, and 8 d. All LC-MRM analyses were repeated thrice.

**BUILDING CLASSIFIERS for sample set definition**

A discovery set (N = 300) was used to build an age-related macular degeneration (AMD) classifier. After creating the classifier, we tested their performance on an independent validation set. More specifically, the samples used in the AMD validation set did not overlap nor were they used in the discovery set [^9^](#_ENREF_9).

**MACHINE LEARNING METHOD OF THE CLASSIFIER**

We selected three proteins (insulin-like growth factor binding protein 2 [IGFBP2], selectin E [SELE], and thrombospondin 1 [THBS1]). Prior to model building, centering and scaling to the protein concentration (ng/mL) were performed as pre-processing on the discovery set. To build an AMD classifier, the outcome value was defined as 1 for AMD and 0 for other groups, according to the sample type.

We generated the classifiers fit to support vector machine (SVM) models [^10^](#_ENREF_10) based on multiple peptide concentrations belonging to each disease panel and subsequently built logistic regression (LR) models [^11^](#_ENREF_11) including SVM prediction value and several clinical factors. In the discovery set, a Gaussian radial kernel SVM model with optimized cost parameters was generated by ten-fold cross-validation with thousand repeats. An SVM model with a radial basis kernel function was built using the R package “kernlab” in the caret interface [^12^](#_ENREF_12). Model parameters were optimized through repeated (1,000 iterations) 10-fold cross-validation of the MRM data of the discovery set. The outcomes of the SVM classifier for each sample were derived as disease prediction scores between zero and one. The AMD-SVM model with a Gaussian radial kernel was generated by a 1,000 repeated ten-fold cross-validation method (parameter sigma = 1.21 and C = 4). Subsequently, we applied the training process to build an LR model, including the SVM outcome (0–1 probability) and clinical factors, as follows. Age was a numeric variable, smoking was a nominal variable, body mass index was a numeric variable, hypertension was a nominal variable, hyperlipidemia was a nominal variable, and the two CFH variant ratios were nominal variables. The nominal variable smoking was defined as 0 as a non-smoker, 0.5 as an ex-smoker, and 1 as a smoker. Nominal hypertension variables were defined as 1 for hypertension and 0 for non-hypertension. The hyperlipidemia nominal variables were defined as 1 for hyperlipidemia and 0 for non-hyperlipidemia. For rs800292:p.Val62Ile, the first allele of the two CFH variants was defined as 1 when the concentration ratio of SLGNVIMVCR to SLGNIIMVCR was less than 0.1, 0.5 when between 0.1 and 15, and 1 when it was greater than 15. For rs1061170:p.Tyr402His, the second allele of the two CFH variants was defined as 1 when the concentration ratio of CYFPYLENGYNQNYGR to CYFPYLENGYNQNHGR was less than 0.1, 0.5 when between 0.1 and 15, and 1 when greater than 15. In the discovery set, LR models with optimized coefficients were generated via ten-fold cross-validation with thousand repeats to use the R package, “kernlab,” in caret interface.

**Data analysis**

Statistical analysis was performed in RStudio (version 1.4.1717), including R software (version 4.1.0), with several packages that contained caret for applying SVM and LR methods, ggplot2 for displaying the violin and volcano plots, and pROC [^13^](#_ENREF_13) to display AUROC curves and calculate the sensitivity and specificity. For reverse response curve plotting and analytical validation, the mean and standard deviation were calculated using Microsoft Excel (ver. 2202).

**ANIMAL EXPERIMENTS USING MOUSE MODELS FOR AGING AND AMD**

Animal care and experimental procedures were performed with the approval of the Animal Care Committee of Asan Medical Center. Mice were handled in accordance with the ARVO Statement for the Use of Animals in Ophthalmic and Vision Research. Specific pathogen-free C57BL/6J mice. One-year-old mice were used as an old mice group. Eight-week-old mice were controls. For the neovascular AMD model, the laser-induced choroidal neovascularization (LiCNV) in mice was generated by a previously described method [^14^](#_ENREF_14). Male C57BL/6J mice were anesthetized with an intramuscular injection of 40 mg/kg ketamine and 12 mg/kg xylazine. Topical 0.5% proparacaine was applied, and the pupils were dilated with 2.5% phenylephrine (Alcon Fort Worth, TX). A laser photocoagulator (Carl Zeiss, Oberkochen, Germany) with a slit lamp delivery system was used with a coverslip as a contact lens to visualize the retina. Sufficient laser energy (250 mW, 100 ms, 50 μm) was delivered to rupture Bruch’s membrane in three locations for each eye (the 2, 6, and 10 o’clock positions of the posterior pole). The CNV formation was confirmed by the production of a bubble at the time of laser photocoagulation, which indicates the rupture of Bruch’s membrane, an essential factor in obtaining experimental CNV.

**QUANTITATIVE REAL-TIME POLYMERASE CHAIN REACTION (RT-PCR)**

The total RNA from the retinal cups was extracted using the RNeasy Plus Mini Kit (Qiagen, Hilden, Germany) according to the manufacturer’s instructions and was reverse transcribed into cDNA using the GoScriptTM Reverse Transcription Kit (Promega, Madison, WI, USA). Quantitative RT-PCR was performed using the FastStart SYBR Green Master Mix (Roche, Basel, Switzerland) with the indicated primers using a real-time PCR detection system (Bio-Rad TM CFX96; Bio-Rad Laboratories, Inc., Hercules, CA, USA). The RT-PCR data were analyzed using amplification software (Bio-Rad CFX Manager; Bio-Rad Laboratories, Inc.). GAPDH was used as a reference gene, and the results were presented as the relative expression based on the control.

**REFERENCES**

1 Age-Related Eye Disease Study Research, G. A randomized, placebo-controlled, clinical trial of high-dose supplementation with vitamins C and E, beta carotene, and zinc for age-related macular degeneration and vision loss: AREDS report no. 8. *Arch Ophthalmol* **119**, 1417-1436, doi:10.1001/archopht.119.10.1417 (2001).

2 Kim, H. J. *et al.* Identification of vinculin as a potential plasma marker for age-related macular degeneration. *Invest Ophthalmol Vis Sci* **55**, 7166-7176, doi:10.1167/iovs.14-15168 (2014).

3 Ahn, H. S., Park, S. J., Jung, H. G., Woo, S. J. & Lee, C. Quantification of protein markers monitoring the pre-analytical effect of blood storage time before plasma isolation using (15) N metabolically labeled recombinant proteins. *J Mass Spectrom* **53**, 1189-1197, doi:10.1002/jms.4294 (2018).

4 Rai, A. J. *et al.* HUPO Plasma Proteome Project specimen collection and handling: towards the standardization of parameters for plasma proteome samples. *Proteomics* **5**, 3262-3277, doi:10.1002/pmic.200401245 (2005).

5 Abbatiello, S. E. *et al.* Design, implementation and multisite evaluation of a system suitability protocol for the quantitative assessment of instrument performance in liquid chromatography-multiple reaction monitoring-MS (LC-MRM-MS). *Mol Cell Proteomics* **12**, 2623-2639, doi:10.1074/mcp.M112.027078 (2013).

6 Carr, S. A. *et al.* Targeted peptide measurements in biology and medicine: best practices for mass spectrometry-based assay development using a fit-for-purpose approach. *Mol Cell Proteomics* **13**, 907-917, doi:10.1074/mcp.M113.036095 (2014).

7 Addona, T. A. *et al.* Multi-site assessment of the precision and reproducibility of multiple reaction monitoring-based measurements of proteins in plasma. *Nat Biotechnol* **27**, 633-641, doi:10.1038/nbt.1546 (2009).

8 Alankar Shrivastava, V. B. G. Methods for the determination of limit of detection and limit of quantitation of the analytical methods. *Chronicles of Young Scientists* **2**, 21-25 (2011).

9 Jung, Y. & Hu, J. A K-fold Averaging Cross-validation Procedure. *J Nonparametr Stat* **27**, 167-179, doi:10.1080/10485252.2015.1010532 (2015).

10 Cortes, C. & Vapnik, V. Support-vector networks. *Machine learning* **20**, 273-297 (1995).

11 Cox, D. R. The Regression Analysis of Binary Sequences. *Journal of the Royal Statistical Society. Series B (Methodological)* **20**, 215-242 (1958).

12 Kuhn, M. Building Predictive Models in R Using the caret Package. *Journal of Statistical Software* **28**, 1 - 26, doi:10.18637/jss.v028.i05 (2008).

13 Robin, X. *et al.* pROC: an open-source package for R and S+ to analyze and compare ROC curves. *BMC Bioinformatics* **12**, 77, doi:10.1186/1471-2105-12-77 (2011).

14 Lee, J. *et al.* Angiopoietin-1 suppresses choroidal neovascularization and vascular leakage. *Invest Ophthalmol Vis Sci* **55**, 2191-2199, doi:10.1167/iovs.14-13897 (2014).
