# Supplementary material for: Multiplexed plasma protein classifiers for the diagnosis of age‐related macular degeneration
Source: Clin Transl Med. 2023 Jun 14;13(6):e1307. doi: 10.1002/ctm2.1307 (PMC10267425; doi:10.1002/ctm2.1307)
Supplement: Supplementary file 2 — Supplementary Information [file CTM2-13-e1307-s011.docx]

**
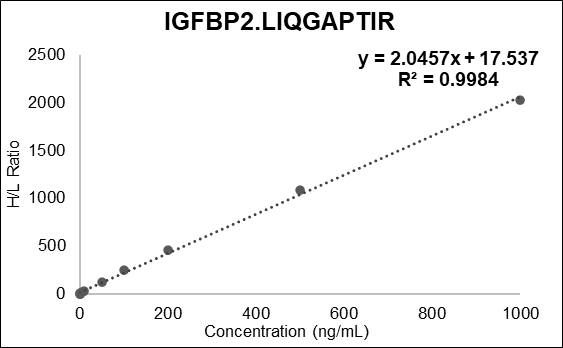

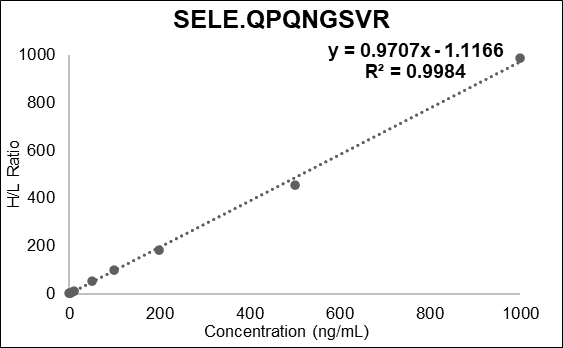

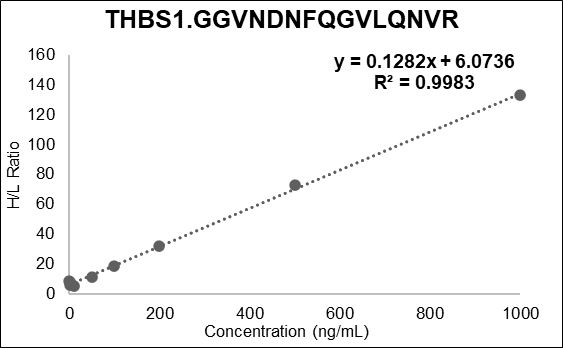

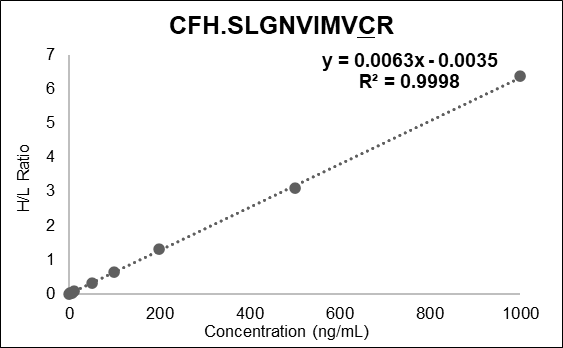

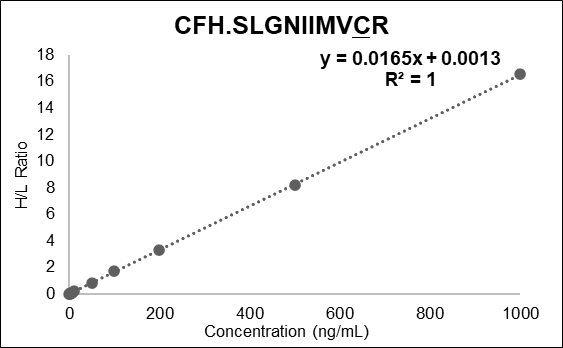

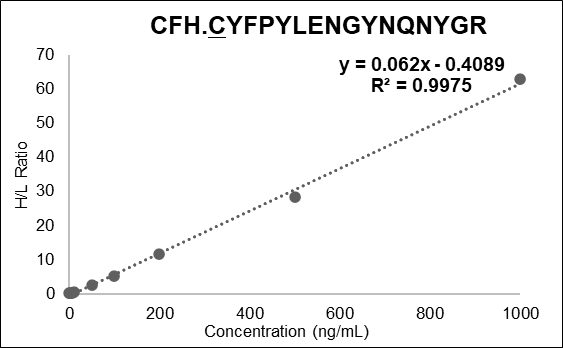

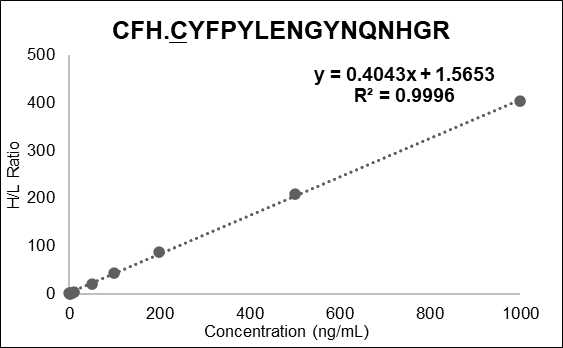
**

**Figure S1. Calibration curves of seven peptides based on heavy to light peptide extracted ion chromatogram ratio.**

The linearity of the multiple reaction monitoring (MRM)-mass spectrometry (MS) assay was determined by spiking the stable isotope-labeled internal standard peptides into plasma at various concentrations (0.1–1,000 fmol). A plot (ng/mL) of the ratio of heavy concentration to light peak area calculated from the average of three replicates of seven peptides is shown. A linear regression equation for each peptide is shown, and the R^2^ value was 0.99 or higher. **C**: Carbamidomethyl cysteine.
