# Supplementary material for: Multiplexed plasma protein classifiers for the diagnosis of age‐related macular degeneration
Source: Clin Transl Med. 2023 Jun 14;13(6):e1307. doi: 10.1002/ctm2.1307 (PMC10267425; doi:10.1002/ctm2.1307)
Supplement: Supplementary file 3 — Supplementary Information [file CTM2-13-e1307-s003.docx]

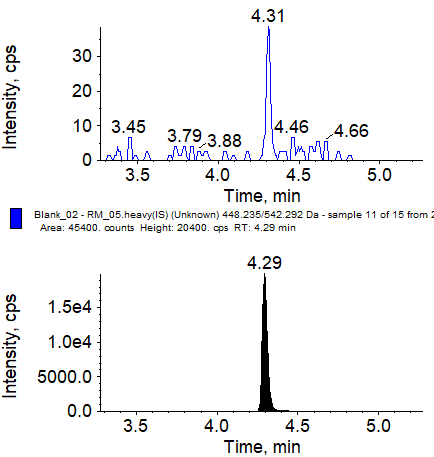

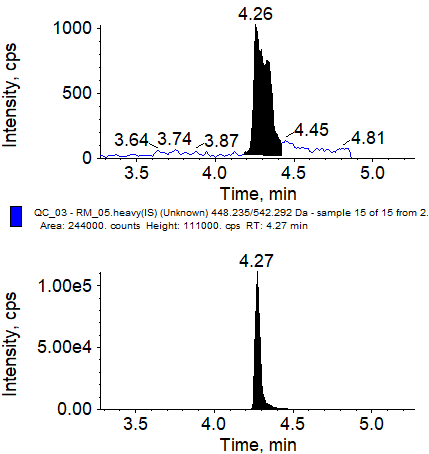

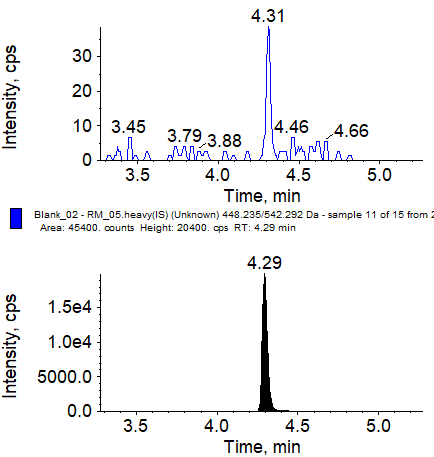


**SELE.QPQNGSVR**

**Heavy**

**Heavy**


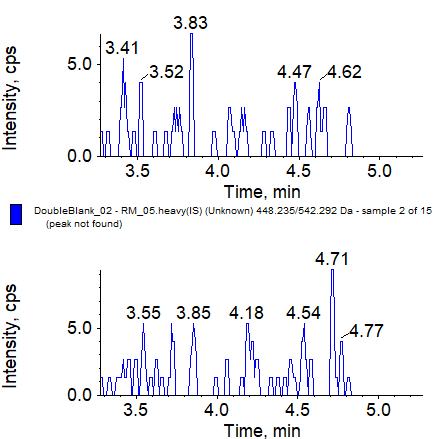


**Light**


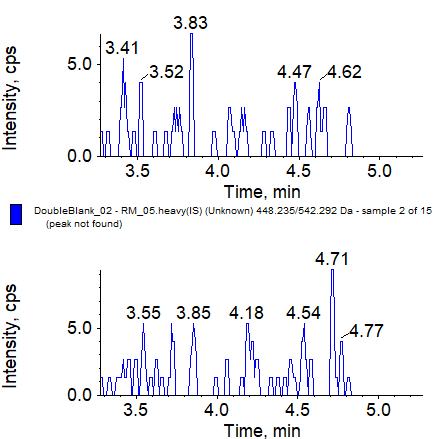


**Heavy**

**Light**


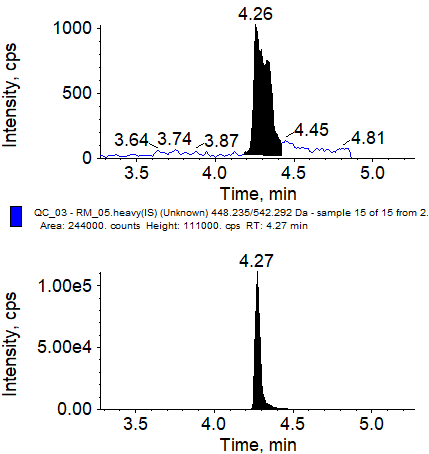


**Light**

**Double blank**

**Specificity analyte**

**Low-QC sample**


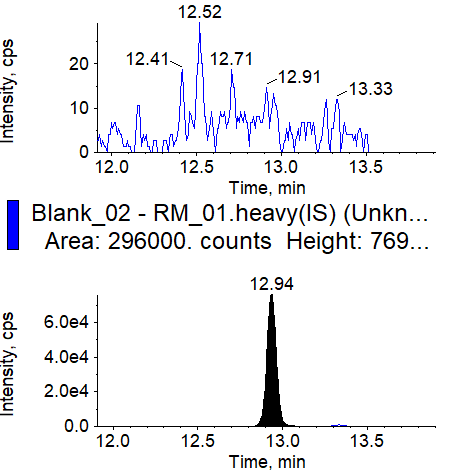

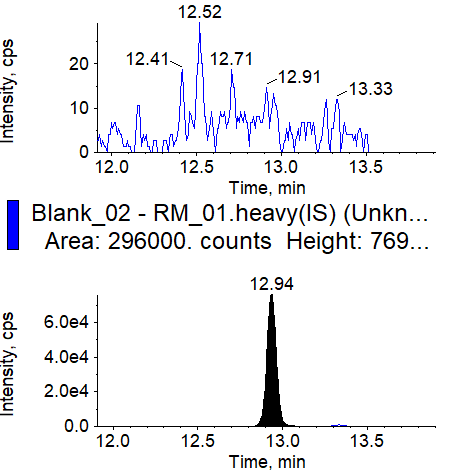


**IGFBP2.LIQGAPTIR**

**Heavy**


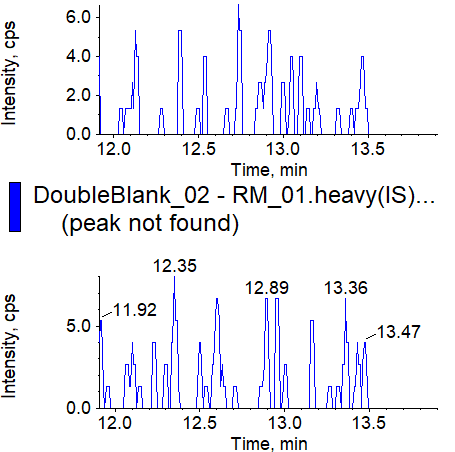


**Light**


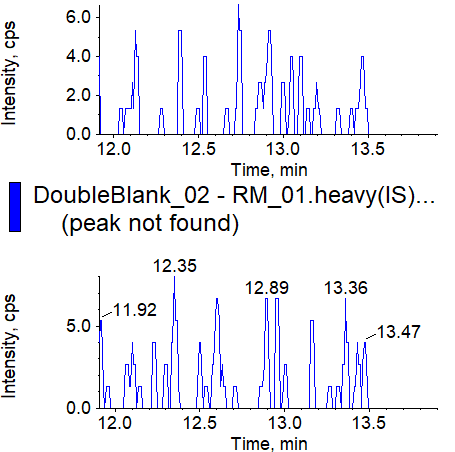


**Heavy**

**Light**


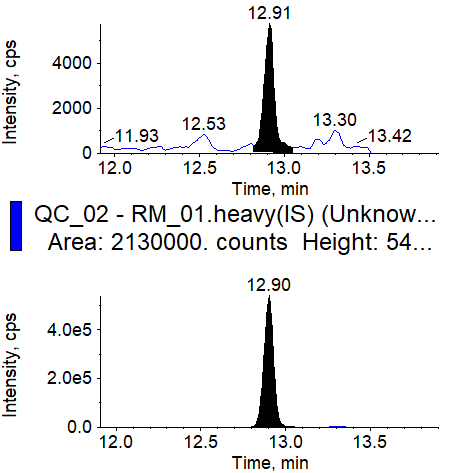

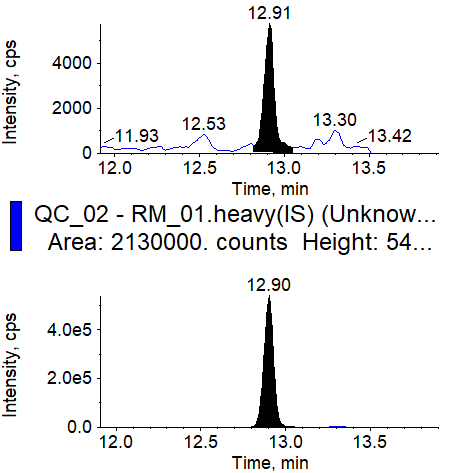


**Light**

**Heavy**


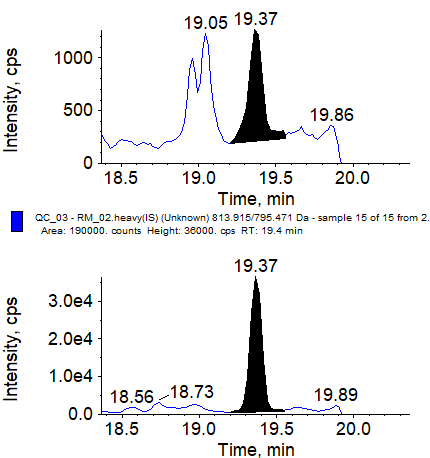

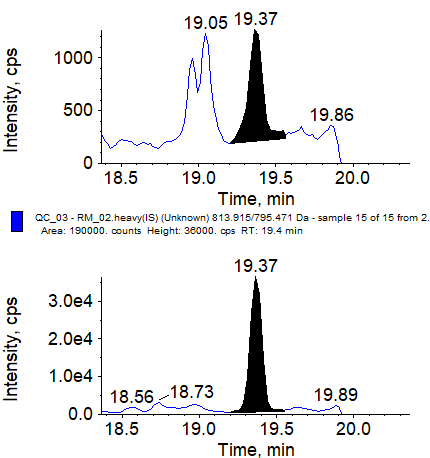

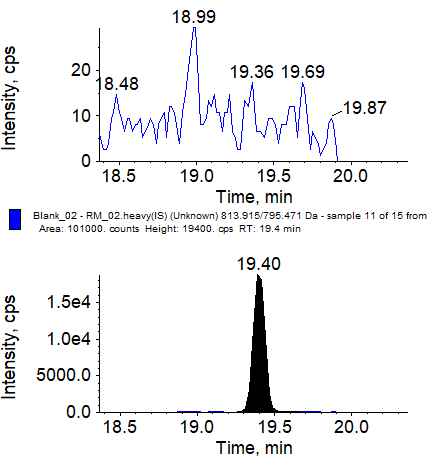


**THBS1.GGVNDNFQGVLQNVR**

**Heavy**

**Light**

**Heavy**


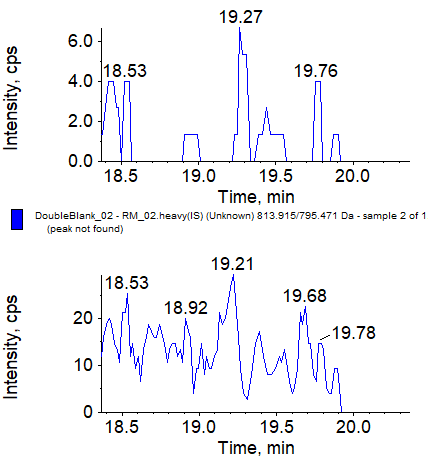

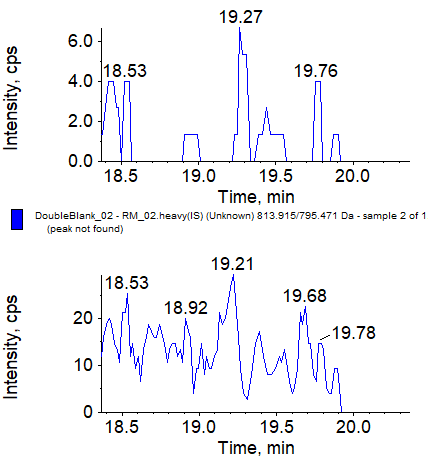


**Heavy**

**Light**


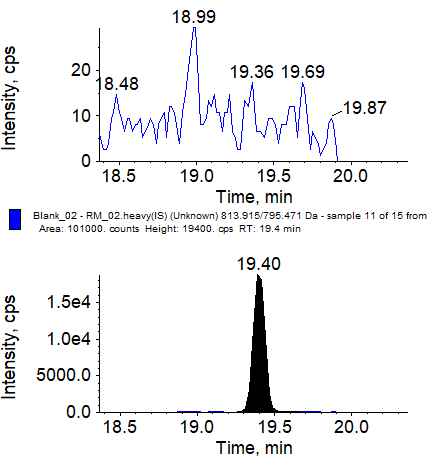


**Light**


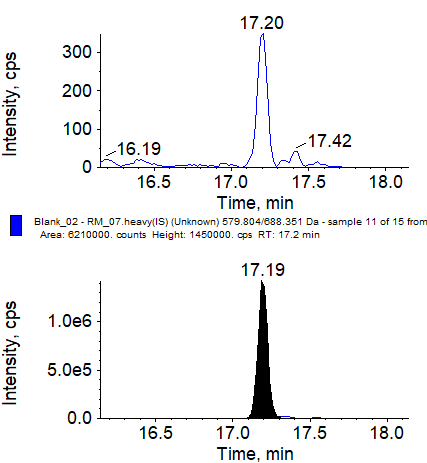

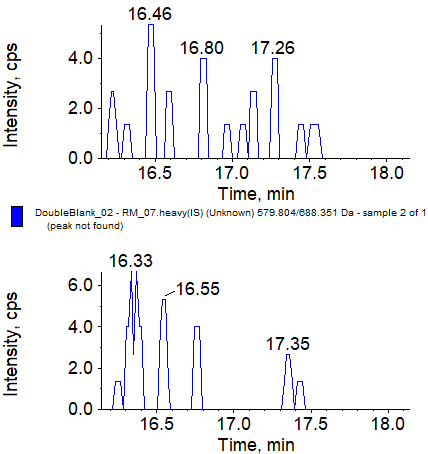

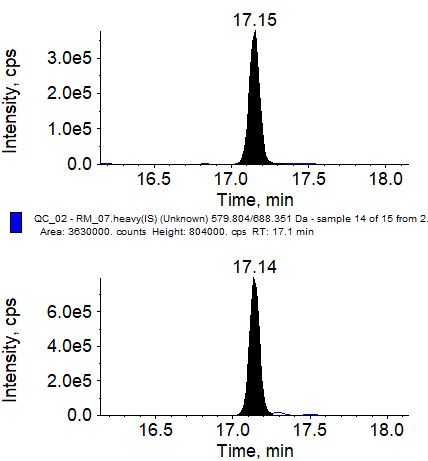

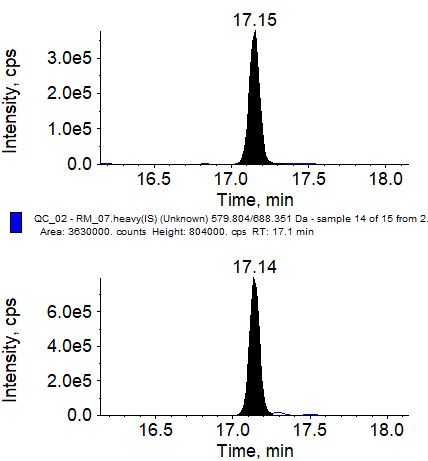

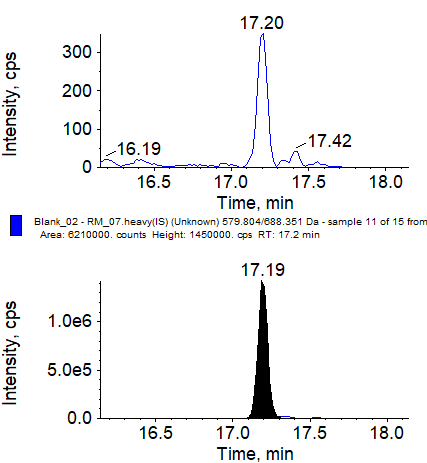


**CFH.SLGNVIMVCR**

**Light**

**Heavy**

**Light**

**Heavy**


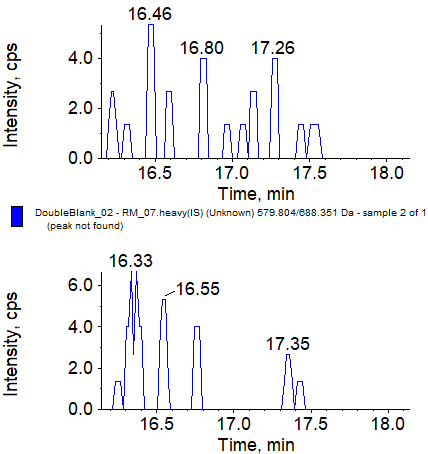


**Light**

**Heavy**


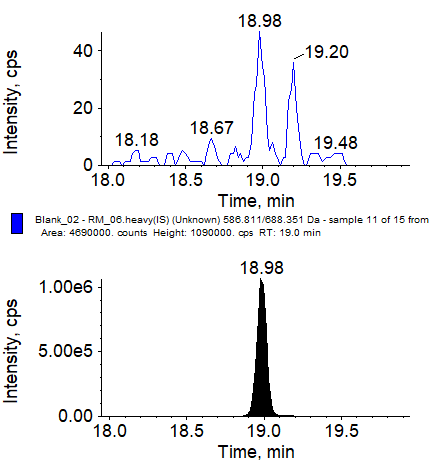

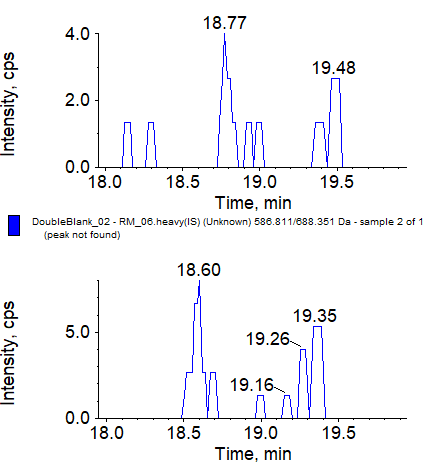

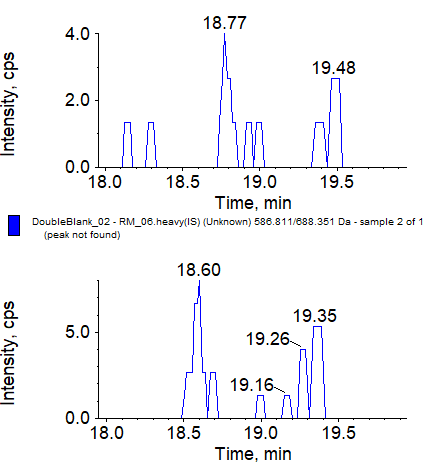

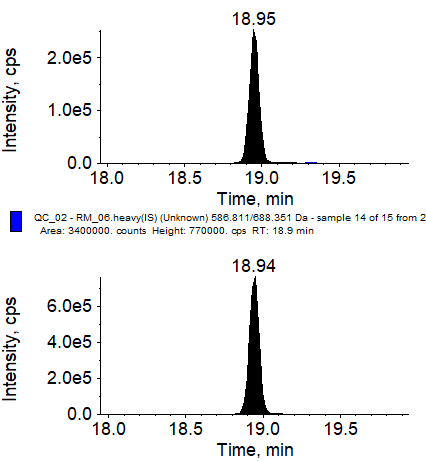

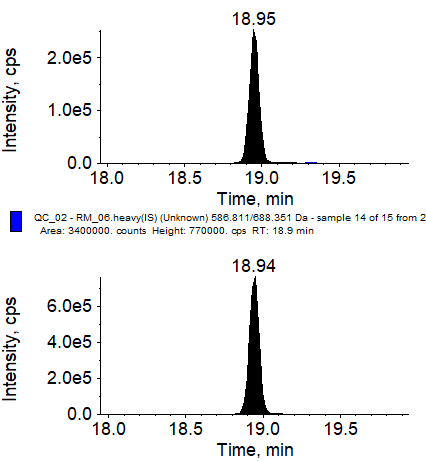

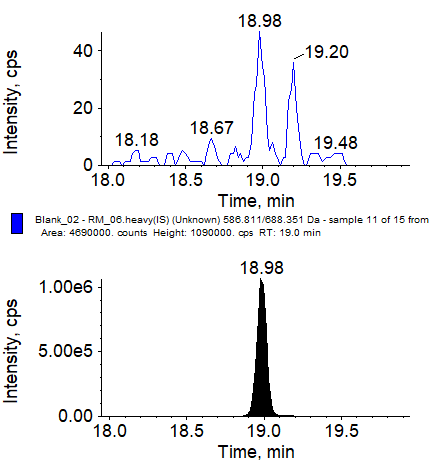


**CFH.SLGNIIMVCR**

**Light**

**Heavy**

**Light**

**Heavy**

**Light**

**Heavy**


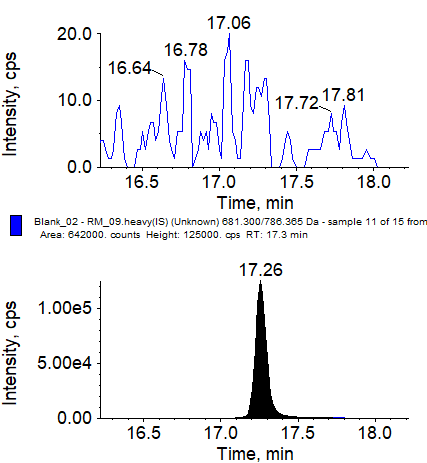

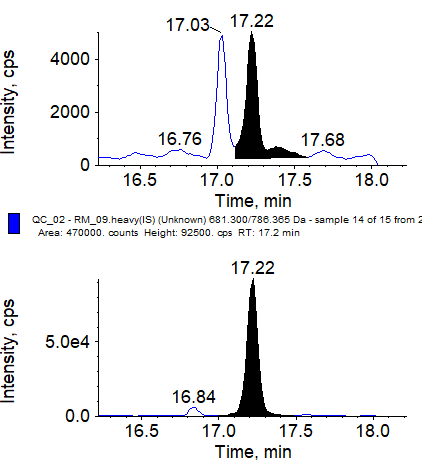

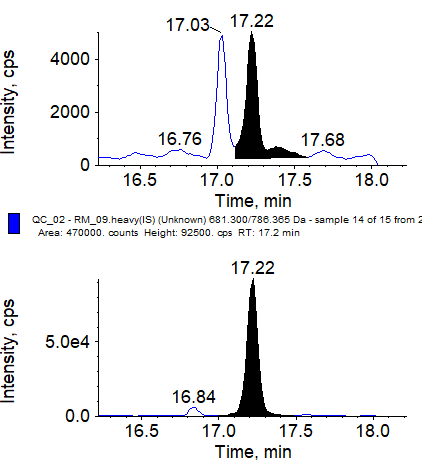

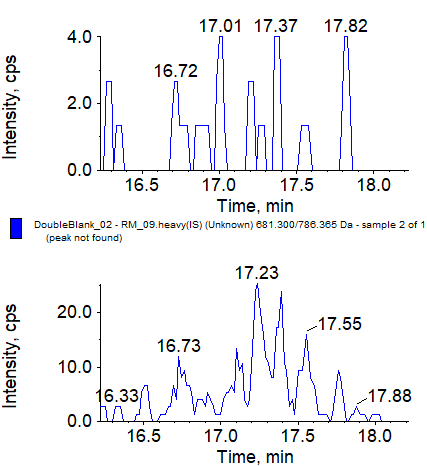

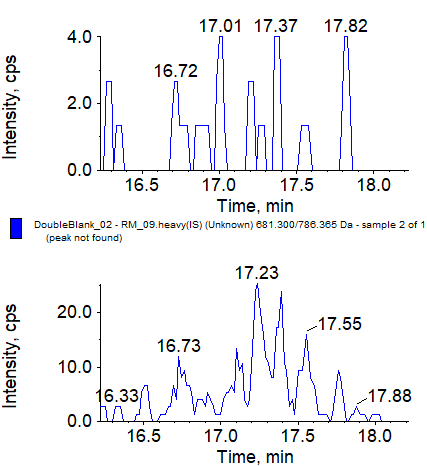

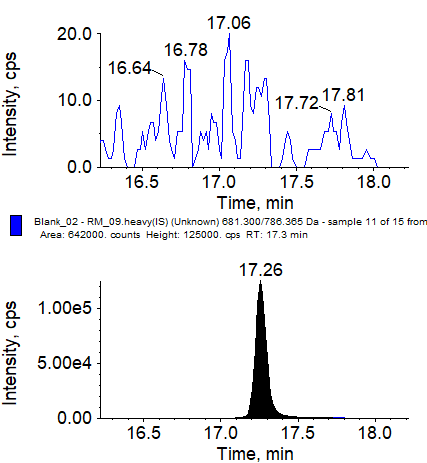


**CFH.CYFPYLENGYNQNHGR**

**Light**

**Heavy**

**Light**

**Heavy**

**Light**

**Heavy**


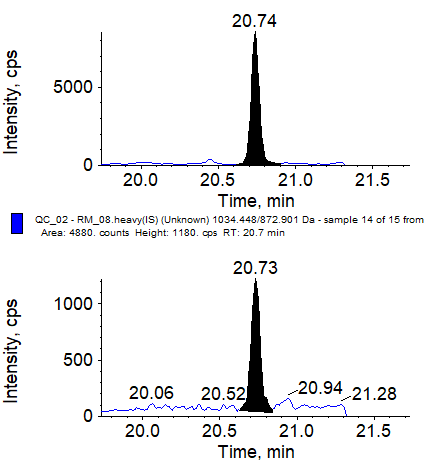

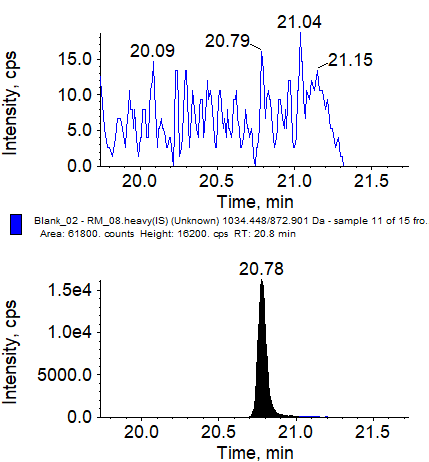

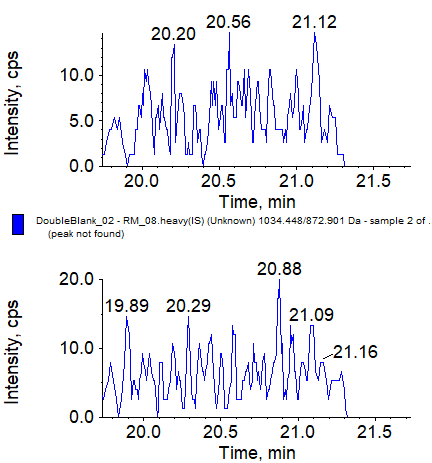

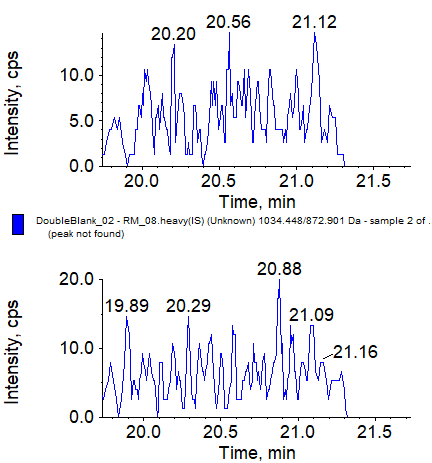

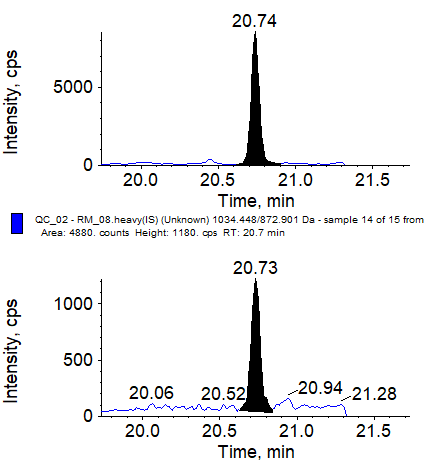

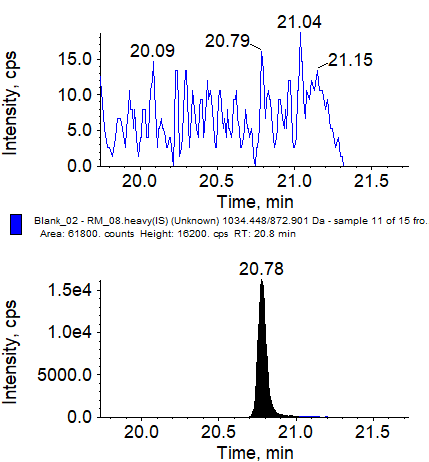


**CFH.CYFPYLENGYNQNYGR**

**Light**

**Heavy**

**Light**

**Heavy**

**Light**

**Heavy**

**Double blank**

**Specificity analyte**

**Low-QC sample**

**Figure S2. Evaluation of the specificities of seven peptides.**

In the specificity analysis, seven peptides were not detected in the six double blanks. Only the standard substances were detected in the blank sample. In addition, only endogenous substances were detected in specific analytes, which are endogenous materials without standards, and both endogenous substances and standard substances were detected in low-quality control (QC) samples.
