# Supplementary material for: Multiplexed plasma protein classifiers for the diagnosis of age‐related macular degeneration
Source: Clin Transl Med. 2023 Jun 14;13(6):e1307. doi: 10.1002/ctm2.1307 (PMC10267425; doi:10.1002/ctm2.1307)
Supplement: Supplementary file 4 — Supplementary Information [file CTM2-13-e1307-s006.docx]

**Figure S3. Performance of the** **age-related macular degeneration (AMD) classifiers in the corresponding discovery set.** The set consisted of 180 control participants and 120 AMD participants. The area under the receiver operating characteristic (AUROC) curve is 0.876 (95% confidence interval: 0.836-0.915). AUROC analysis was performed using the R package (“pROC”).
