# Supplementary material for: Multiplexed plasma protein classifiers for the diagnosis of age‐related macular degeneration
Source: Clin Transl Med. 2023 Jun 14;13(6):e1307. doi: 10.1002/ctm2.1307 (PMC10267425; doi:10.1002/ctm2.1307)
Supplement: Supplementary file 5 — Supplementary Information [file CTM2-13-e1307-s009.docx]

**Figure S4. Violin with boxplots of age-related macular degeneration (AMD) prediction scores in the two groups for the discovery set and validation set.** The discovery set consisted of 180 control subjects and 120 AMD subjects (A). The validation set consisted of 393 control subjects and 220 AMD subjects (B).
