# Supplementary material for: Multiplexed plasma protein classifiers for the diagnosis of age‐related macular degeneration
Source: Clin Transl Med. 2023 Jun 14;13(6):e1307. doi: 10.1002/ctm2.1307 (PMC10267425; doi:10.1002/ctm2.1307)
Supplement: Supplementary file 6 — Supplementary Information [file CTM2-13-e1307-s001.docx]

**
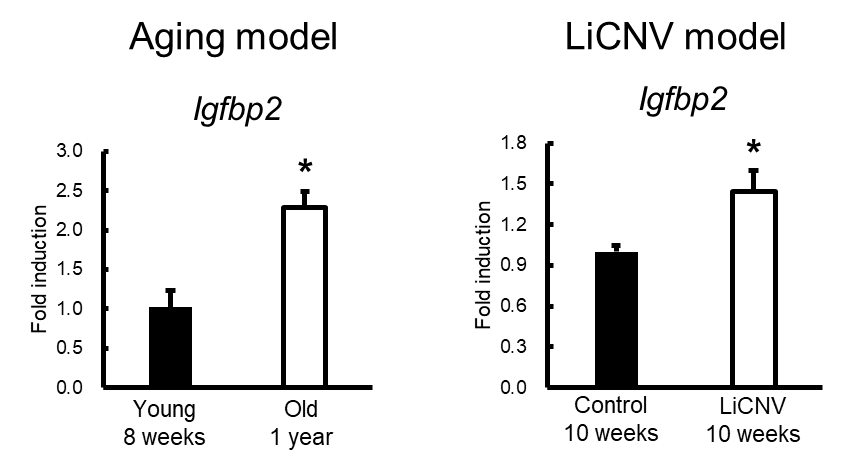
**

**Figure S5. mRNA expression level of *Igfbp2* in the retinal of aged mice and a laser-induced choroidal neovascularization (LiCNV) mice.** LiCNV mice were exposed to laser light and then retinal was extracted 14 days later. Each group N=4, *P<0.05.
