# Supplementary material for: Multiplexed plasma protein classifiers for the diagnosis of age‐related macular degeneration
Source: Clin Transl Med. 2023 Jun 14;13(6):e1307. doi: 10.1002/ctm2.1307 (PMC10267425; doi:10.1002/ctm2.1307)
Supplement: Supplementary file 7 — Supplementary Information [file CTM2-13-e1307-s007.docx]

**Table S1. Clinical characteristics of participants in the discovery and validation studies.**

| **Variables** | **Discovery Set**  **(n = 300)** | | **Validation Set**  **(n = 613)** | |
| --- | --- | --- | --- | --- |
|  | **Control  (*n* = 180)** | **AMD  (*n* = 120)** | **Control**  **(n = 393)** | **AMD  (n = 220)** |
| Age (years) | 62.28 ± 8.37 | 69.96 ± 8.09 | 58.39 ± 7.68 | 70.07 ± 7.50 |
| Gender (F/M) | 1.02 | 1 | 1.1 | 1.3 |
| BMI | 24.24 ± 3.35 | 23.76 ± 3.04 | 24.05 ± 2.94 | 23.96 ± 2.94 |
| Subtype |  |  |  |  |
| Early AMD | − | 55 | − | 85 |
| Late AMD | − | 65 | − | 135 |
| Systemic risk factors |  |  |  |  |
| Smoking |  |  |  |  |
| Current smoker | 25 | 7 | 50 | 14 |
| Ex-smoker | 72 | 44 | 118 | 71 |
| Non-smoker | 83 | 69 | 225 | 135 |
| Hypertension | 99 | 65 | 129 | 105 |
| Hyperlipidemia | 82 | 45 | 134 | 87 |
| Cohort |  |  |  |  |
| SNUBH | 180 | 120 | 328 | 100 |
| AMC | − | − | 65 | 120 |

AMD, age-related macular degeneration; BMI, body mass index; AMC Asan Medical Center; SNUBH Seoul National University Bundang Hospital.
