# Supplementary material for: Multiplexed plasma protein classifiers for the diagnosis of age‐related macular degeneration
Source: Clin Transl Med. 2023 Jun 14;13(6):e1307. doi: 10.1002/ctm2.1307 (PMC10267425; doi:10.1002/ctm2.1307)
Supplement: Supplementary file 8 — Supplementary Information [file CTM2-13-e1307-s012.docx]

**Table S2. Calibration curve results of seven peptides used for MRM-MS analysis.**

| **No.** | **Protein/peptide** | **Heavy Conc. (ng/mL)** | **H/L Area Ratio**  **(1^st^ Run)** | **H/L Area Ratio**  **(2^nd^ Run)** | **H/L Area Ratio**  **(3^rd^ Run)** | **Mean** | **SD** | **CV (%)** | **Linearity (R^2^)** | **LOD (cps)** | **LOQ (cps)** |
| --- | --- | --- | --- | --- | --- | --- | --- | --- | --- | --- | --- |
| 1 | IGFBP2/  LIQGAPTIR | 0.1 | 0.435 | 0.392 | 0.396 | 0.408 | 0.023 | 5.739 | 0.9984 | 1302 | 4340 |
|  |  | 0.5 | 1.241 | 1.344 | 1.223 | 1.269 | 0.065 | 5.147 |  |  |  |
|  |  | **1.0** | **2.816** | **2.510** | **2.662** | **2.663** | **0.153** | **5.756** |  |  |  |
|  |  | 5.0 | 12.560 | 13.455 | 13.401 | 13.139 | 0.502 | 3.819 |  |  |  |
|  |  | 10.0 | 25.960 | 25.243 | 26.244 | 25.815 | 0.516 | 1.998 |  |  |  |
|  |  | 50.0 | 121.395 | 121.531 | 129.534 | 124.153 | 4.660 | 3.753 |  |  |  |
|  |  | 100.0 | 251.442 | 240.201 | 256.566 | 249.403 | 8.371 | 3.356 |  |  |  |
|  |  | 200.0 | 474.396 | 415.982 | 483.721 | 458.033 | 36.715 | 8.016 |  |  |  |
|  |  | 500.0 | 1088.785 | 1074.766 | 1103.627 | 1089.059 | 14.432 | 1.325 |  |  |  |
|  |  | 1000.0 | 2031.250 | 2053.398 | 2004.902 | 2029.850 | 24.278 | 1.196 |  |  |  |
| 2 | SELE/  QPQNGSVR | 0.1 | 2.390 | 1.888 | 1.876 | 2.051 | 0.293 | 14.298 | 0.9984 | 2340 | 7800 |
|  |  | 0.5 | 2.502 | 2.428 | 2.262 | 2.397 | 0.123 | 5.130 |  |  |  |
|  |  | 1.0 | 2.687 | 2.643 | 2.264 | 2.531 | 0.232 | 9.176 |  |  |  |
|  |  | **5.0** | **7.153** | **7.087** | **6.946** | **7.062** | **0.106** | **1.495** |  |  |  |
|  |  | 10.0 | 11.527 | 9.878 | 10.917 | 10.774 | 0.834 | 7.738 |  |  |  |
|  |  | 50.0 | 47.268 | 58.075 | 54.381 | 53.241 | 5.493 | 10.317 |  |  |  |
|  |  | 100.0 | 100.000 | 92.938 | 109.732 | 100.890 | 8.432 | 8.358 |  |  |  |
|  |  | 200.0 | 190.606 | 178.465 | 175.719 | 181.597 | 7.922 | 4.363 |  |  |  |
|  |  | 500.0 | 449.438 | 487.805 | 425.170 | 454.138 | 31.581 | 6.954 |  |  |  |
|  |  | 1000.0 | 942.559 | 1037.500 | 978.395 | 986.151 | 47.944 | 4.862 |  |  |  |
| 3 | THBS1/  GGVNDNFQGVLQNVR | 0.1 | 8.655 | 8.637 | 8.113 | 8.468 | 0.308 | 3.638 | 0.9983 | 276 | 920 |
|  |  | 0.5 | 6.560 | 8.644 | 8.396 | 7.866 | 1.138 | 14.471 |  |  |  |
|  |  | 1.0 | 4.873 | 5.585 | 5.967 | 5.475 | 0.555 | 10.139 |  |  |  |
|  |  | 5.0 | 4.442 | 6.147 | 7.732 | 6.107 | 1.645 | 26.943 |  |  |  |
|  |  | 10.0 | 4.646 | 5.303 | 4.550 | 4.833 | 0.410 | 8.485 |  |  |  |
|  |  | **50.0** | **9.851** | **12.340** | **10.676** | **10.956** | **1.268** | **11.576** |  |  |  |
|  |  | 100.0 | 18.017 | 17.554 | 20.412 | 18.661 | 1.534 | 8.220 |  |  |  |
|  |  | 200.0 | 29.252 | 35.103 | 31.209 | 31.855 | 2.978 | 9.350 |  |  |  |
|  |  | 500.0 | 77.685 | 67.596 | 72.836 | 72.706 | 5.045 | 6.940 |  |  |  |
|  |  | 1000.0 | 137.543 | 131.153 | 130.619 | 133.105 | 3.853 | 2.894 |  |  |  |
| 4 | CFH/  SLGNVIMV**C**R | 0.1 | 0.003 | 0.003 | 0.003 | 0.003 | 0.000 | 1.420 | 0.9998 | 793 | 2643 |
|  |  | 0.5 | 0.005 | 0.005 | 0.005 | 0.005 | 0.000 | 2.184 |  |  |  |
|  |  | 1.0 | 0.008 | 0.008 | 0.008 | 0.008 | 0.000 | 0.657 |  |  |  |
|  |  | **5.0** | **0.032** | **0.029** | **0.031** | **0.031** | **0.001** | **4.554** |  |  |  |
|  |  | 10.0 | 0.064 | 0.068 | 0.066 | 0.066 | 0.002 | 3.192 |  |  |  |
|  |  | 50.0 | 0.325 | 0.307 | 0.303 | 0.312 | 0.011 | 3.650 |  |  |  |
|  |  | 100.0 | 0.627 | 0.604 | 0.630 | 0.620 | 0.014 | 2.238 |  |  |  |
|  |  | 200.0 | 1.280 | 1.305 | 1.308 | 1.298 | 0.015 | 1.163 |  |  |  |
|  |  | 500.0 | 3.050 | 3.113 | 3.085 | 3.082 | 0.031 | 1.016 |  |  |  |
|  |  | 1000.0 | 6.625 | 6.358 | 6.119 | 6.368 | 0.253 | 3.971 |  |  |  |
| 5 | †CFH/  SLGNIIMV**C**R | 0.1 | 0.006 | 0.006 | 0.006 | 0.006 | 0.000 | 1.865 | 1.000 | 340 | 1132 |
|  |  | 0.5 | 0.011 | 0.011 | 0.010 | 0.010 | 0.000 | 1.885 |  |  |  |
|  |  | 1.0 | 0.017 | 0.018 | 0.018 | 0.017 | 0.001 | 3.320 |  |  |  |
|  |  | **5.0** | **0.080** | **0.080** | **0.079** | **0.080** | **0.000** | **0.250** |  |  |  |
|  |  | 10.0 | 0.163 | 0.166 | 0.172 | 0.167 | 0.005 | 2.754 |  |  |  |
|  |  | 50.0 | 0.763 | 0.825 | 0.818 | 0.802 | 0.034 | 4.265 |  |  |  |
|  |  | 100.0 | 1.686 | 1.745 | 1.776 | 1.736 | 0.046 | 2.636 |  |  |  |
|  |  | 200.0 | 3.158 | 3.257 | 3.374 | 3.263 | 0.108 | 3.307 |  |  |  |
|  |  | 500.0 | 8.554 | 7.879 | 8.184 | 8.206 | 0.339 | 4.125 |  |  |  |
|  |  | 1000.0 | 17.117 | 16.667 | 15.784 | 16.523 | 0.678 | 4.103 |  |  |  |
| 6 | CFH/  **C**YFPYLENGYNQNYGR | 0.1 | 0.083 | 0.117 | 0.157 | 0.119 | 0.037 | 31.217 | 0.9976 | 107 | 357 |
|  |  | 0.5 | 0.063 | 0.079 | 0.070 | 0.070 | 0.008 | 10.830 |  |  |  |
|  |  | 1.0 | 0.081 | 0.080 | 0.079 | 0.080 | 0.001 | 1.395 |  |  |  |
|  |  | **5.0** | **0.300** | **0.276** | **0.299** | **0.292** | **0.014** | **4.680** |  |  |  |
|  |  | 10.0 | 0.559 | 0.489 | 0.533 | 0.527 | 0.035 | 6.734 |  |  |  |
|  |  | 50.0 | 2.514 | 2.512 | 2.667 | 2.564 | 0.089 | 3.460 |  |  |  |
|  |  | 100.0 | 5.479 | 5.245 | 5.210 | 5.311 | 0.146 | 2.745 |  |  |  |
|  |  | 200.0 | 11.273 | 12.021 | 11.454 | 11.582 | 0.390 | 3.368 |  |  |  |
|  |  | 500.0 | 27.023 | 27.841 | 29.660 | 28.175 | 1.350 | 4.791 |  |  |  |
|  |  | 1000.0 | 60.738 | 62.037 | 66.084 | 62.953 | 2.788 | 4.429 |  |  |  |
| 7 | ‡CFH/  **C**YFPYLENGYNQNHGR | 0.1 | 0.340 | 0.399 | 0.467 | 0.402 | 0.064 | 15.884 | 0.9996 | 37 | 123 |
|  |  | 0.5 | 0.322 | 0.268 | 0.332 | 0.308 | 0.034 | 11.203 |  |  |  |
|  |  | 1.0 | 0.442 | 0.408 | 0.389 | 0.413 | 0.027 | 6.559 |  |  |  |
|  |  | **5.0** | **2.092** | **2.108** | **2.238** | **2.146** | **0.080** | **3.715** |  |  |  |
|  |  | 10.0 | 4.591 | 4.215 | 4.232 | 4.346 | 0.213 | 4.891 |  |  |  |
|  |  | 50.0 | 20.273 | 21.196 | 22.024 | 21.164 | 0.876 | 4.138 |  |  |  |
|  |  | 100.0 | 46.789 | 43.360 | 42.588 | 44.246 | 2.236 | 5.054 |  |  |  |
|  |  | 200.0 | 85.311 | 87.228 | 87.172 | 86.570 | 1.091 | 1.260 |  |  |  |
|  |  | 500.0 | 204.507 | 212.784 | 206.723 | 208.005 | 4.285 | 2.060 |  |  |  |
|  |  | 1000.0 | 401.685 | 388.601 | 417.867 | 402.718 | 14.660 | 3.640 |  |  |  |

Conc., Concentration; H/L, heavy to light; CPS, counts per second; SD, standard deviation; CV, coefficient of variance; **C**, carbamidomethyl-cysteine; †CFH, rs800292 (Val62Ile); ‡CFH, rs1061170 (Tyr402His).
