# Supplementary material for: Multiplexed plasma protein classifiers for the diagnosis of age‐related macular degeneration
Source: Clin Transl Med. 2023 Jun 14;13(6):e1307. doi: 10.1002/ctm2.1307 (PMC10267425; doi:10.1002/ctm2.1307)
Supplement: Supplementary file 9 — Supplementary Information [file CTM2-13-e1307-s010.docx]

**Table S3. Analytical stability test results for seven peptides.**

| **Protein/peptide** | **Sample** | **Different time points** | | | | | | | | **Mean (ng/mL)** | **SD** | **CV (%)** |
| --- | --- | --- | --- | --- | --- | --- | --- | --- | --- | --- | --- | --- |
|  |  | **Initial (ng/mL)** | **6 h (ng/mL)** | **12 h (ng/mL)** | **Day 1 (ng/mL)** | **Day 2 (ng/mL)** | **Day 3 (ng/mL)** | **Day 6 (ng/mL)** | **Day 8 (ng/mL)** |  |  |  |
| IGFBP2/  LIQGAPTIR | S1 | 0.1 | 0.2 | 0.1 | 0.2 | 0.2 | 0.1 | 0.2 | 0.2 | 0.2 | 0.0 | 9.5 |
|  | S2 | 0.2 | 0.2 | 0.2 | 0.2 | 0.2 | 0.2 | 0.2 | 0.2 | 0.2 | 0.0 | 7.3 |
|  | S3 | 0.4 | 0.5 | 0.4 | 0.4 | 0.4 | 0.5 | 0.4 | 0.5 | 0.4 | 0.0 | 7.9 |
|  | S4 | 0.3 | 0.3 | 0.3 | 0.3 | 0.3 | 0.3 | 0.3 | 0.3 | 0.3 | 0.0 | 7.4 |
| SELE/  QPQNGSVR | S1 | 0.6 | 0.4 | 0.5 | 0.5 | 0.5 | 0.5 | 0.6 | 0.6 | 0.5 | 0.1 | 13.2 |
|  | S2 | 0.8 | 0.7 | 0.7 | 0.6 | 0.6 | 0.6 | 0.6 | 0.5 | 0.6 | 0.1 | 14.0 |
|  | S3 | 0.5 | 0.7 | 0.6 | 0.7 | 0.6 | 0.7 | 0.7 | 0.7 | 0.6 | 0.1 | 12.2 |
|  | S4 | 0.6 | 0.5 | 0.7 | 0.6 | 0.5 | 0.5 | 0.5 | 0.5 | 0.6 | 0.1 | 13.4 |
| THBS1/  GGVNDNFQGVLQNVR | S1 | 0.0 | 0.0 | 0.0 | 0.0 | 0.0 | 0.0 | 0.0 | 0.0 | 0.0 | 0.0 | 0.0 |
|  | S2 | 4.1 | 4.5 | 5.3 | 4.4 | 4.5 | 5.2 | 4.8 | 4.7 | 4.7 | 0.4 | 8.6 |
|  | S3 | 9.5 | 9.8 | 9.5 | 9.9 | 9.4 | 10.3 | 9.3 | 9.5 | 9.7 | 0.3 | 3.3 |
|  | S4 | 4.6 | 4.4 | 5.0 | 4.2 | 4.6 | 4.4 | 4.9 | 3.4 | 4.4 | 0.5 | 10.7 |
| CFH/  SLGNVIMV**C**R | S1 | 476.8 | 474.5 | 448.6 | 489.9 | 507.5 | 510.9 | 514.7 | 528.6 | 493.9 | 26.3 | 5.3 |
|  | S2 | 262.8 | 237.7 | 249.0 | 244.1 | 244.4 | 227.2 | 241.7 | 223.8 | 241.3 | 12.3 | 5.1 |
|  | S3 | 300.0 | 295.0 | 300.0 | 309.6 | 291.3 | 293.3 | 293.2 | 281.3 | 295.5 | 8.2 | 2.8 |
|  | S4 | 627.9 | 573.2 | 532.3 | 543.8 | 498.5 | 531.2 | 526.6 | 552.7 | 548.3 | 38.7 | 7.1 |
| CFH/  SLGNIIMV**C**R | S1 | 0.0 | 0.0 | 0.0 | 0.0 | 0.0 | 0.0 | 0.0 | 0.0 | 0.0 | 0.0 | 0.0 |
|  | S2 | 263.5 | 257.5 | 261.5 | 271.3 | 274.4 | 264.2 | 265.3 | 267.1 | 265.6 | 5.4 | 2.0 |
|  | S3 | 297.9 | 341.4 | 353.0 | 310.1 | 314.4 | 327.8 | 326.2 | 325.9 | 324.6 | 17.5 | 5.4 |
|  | S4 | 0.0 | 0.0 | 0.0 | 0.0 | 0.0 | 0.0 | 0.0 | 0.0 | 0.0 | 0.0 | 0.0 |
| CFH/  **C**YFPYLENGYNQNYGR | S1 | 37.9 | 38.0 | 33.5 | 33.9 | 45.7 | 43.0 | 47.2 | 34.6 | 39.2 | 5.4 | 13.8 |
|  | S2 | 34.6 | 41.5 | 44.7 | 42.4 | 41.6 | 43.2 | 38.5 | 37.6 | 40.5 | 3.3 | 8.2 |
|  | S3 | 50.4 | 52.9 | 54.9 | 47.2 | 46.4 | 38.9 | 54.8 | 55.9 | 50.2 | 5.8 | 11.5 |
|  | S4 | 46.6 | 46.8 | 49.6 | 38.8 | 44.3 | 47.2 | 45.0 | 45.1 | 45.4 | 3.1 | 6.9 |
| CFH/  **C**YFPYLENGYNQNHGR | S1 | 0.0 | 0.0 | 0.0 | 0.0 | 0.0 | 0.0 | 0.0 | 0.0 | 0.0 | 0.0 | 0.0 |
|  | S2 | 0.0 | 0.0 | 0.0 | 0.0 | 0.0 | 0.0 | 0.0 | 0.0 | 0.0 | 0.0 | 0.0 |
|  | S3 | 0.0 | 0.0 | 0.0 | 0.0 | 0.0 | 0.0 | 0.0 | 0.0 | 0.0 | 0.0 | 0.0 |
|  | S4 | 0.0 | 0.0 | 0.0 | 0.0 | 0.0 | 0.0 | 0.0 | 0.0 | 0.0 | 0.0 | 0.0 |

SD, standard deviation; CV, coefficient of variance; **C**, carbamidomethyl-cysteine.
