# Supplementary material for: Multiplexed plasma protein classifiers for the diagnosis of age‐related macular degeneration
Source: Clin Transl Med. 2023 Jun 14;13(6):e1307. doi: 10.1002/ctm2.1307 (PMC10267425; doi:10.1002/ctm2.1307)
Supplement: Supplementary file 10 — Supplementary Information [file CTM2-13-e1307-s002.docx]

**Table S4. Coefficients of age-related macular degeneration logistic regression classifier.**

| **Variable** | **Coefficient** | **95% CI** | **P** | **SE** |
| --- | --- | --- | --- | --- |
| Intercept | -14.328 | (-19.042, -10.041) | <0.001 | 2.288 |
| PPS_SVM_ | 15.384 | (11.612, 19.575) | <0.001 | 2.024 |
| Age | 0.117 | (0.076, 0.162) | <0.001 | 0.022 |
| Smoking | -0.473 | (-1.452, 0.486) | 0.337 | 0.492 |
| BMI | 0.013 | (-0.087, 0.112) | 0.796 | 0.051 |
| Hypertension | -0.205 | (-0.874, 0.459) | 0.544 | 0.339 |
| Hyperlipidemia | -0.262 | (-0.907, 0.378) | 0.423 | 0.327 |
| CFH-rs800292 | -0.464 | (-1.395, 0.45) | 0.323 | 0.469 |
| CFH-rs1061170 | 0.615 | (-0.936, 2.218) | 0.443 | 0.8 |

95% CI, 95% percentile confidence intervals; P, p-value; SE, standard error; PPS, protein prediction score; BMI, body mass index.
