# Supplementary material for: Multiplexed plasma protein classifiers for the diagnosis of age‐related macular degeneration
Source: Clin Transl Med. 2023 Jun 14;13(6):e1307. doi: 10.1002/ctm2.1307 (PMC10267425; doi:10.1002/ctm2.1307)
Supplement: Supplementary file 11 — Supplementary Information [file CTM2-13-e1307-s005.docx]

**Table S5. Performance of the classifiers in the discovery and validation cohorts at 10% and 15% prevalence of age-related macular degeneration.**

| **Data set** | **Prevalence (%)** | **Threshold** | **Sensitivity (%)** | **Specificity (%)** | **PPV (%)** | **NPV (%)** |
| --- | --- | --- | --- | --- | --- | --- |
| Discovery (n=300) | 10 | 0.44 | 76.6 | 84.4 | 35.4 | 97 |
|  | 15 | 0.29 | 85 | 75 | 37.5 | 96.6 |
| Validation (n=613) | 10 | 0.44 | 65.5 | 84.2 | 31.5 | 95.6 |
|  | 15 | 0.29 | 73.6 | 71.5 | 31.3 | 93.9 |

PPV, positive predictive value; NPV, negative predictive value.
