# Supplementary material for: Multiplexed plasma protein classifiers for the diagnosis of age‐related macular degeneration
Source: Clin Transl Med. 2023 Jun 14;13(6):e1307. doi: 10.1002/ctm2.1307 (PMC10267425; doi:10.1002/ctm2.1307)
Supplement: Supplementary file 12 — Supplementary Information [file CTM2-13-e1307-s008.docx]

**Table S6. Performance of the classifier in two validation cohorts at 10, 15, and 25% prevalence.**

| **Data set** | **Prevalence (%)** | **Threshold** | **Sensitivity (%)** | **Specificity (%)** | **PPV (%)** | **NPV (%)** |
| --- | --- | --- | --- | --- | --- | --- |
| Validation-AMC (n=428) | 10 | 0.44 | 72.5 | 84.6 | 34.4 | 96.5 |
|  | 15 | 0.29 | 77.5 | 67.7 | 29.7 | 94.5 |
|  | 25 | 0.17 | 80.8 | 50.8 | 35.4 | 88.8 |
| Validation-SNUBH (n=185) | 10 | 0.44 | 57 | 84.1 | 28.5 | 94.6 |
|  | 15 | 0.29 | 69 | 72.2 | 30.5 | 93 |
|  | 25 | 0.17 | 73 | 59.1 | 37.3 | 86.8 |

PPV, postive predictive value; NPV, negative predictive value; AMC, Asan Medical Center; SNUBH, Seoul National University of Bundang Hospital.
